# Extraction and Purification of Capsaicin from Capsicum Oleoresin Using a Combination of Tunable Aqueous Polymer-Phase Impregnated Resin (TAPPIR) Extraction and Chromatography Technology

**DOI:** 10.3390/molecules24213956

**Published:** 2019-10-31

**Authors:** Yanmin Lu, Bo Cui

**Affiliations:** State Key Laboratory of Biobased Material and Green Papermaking, School of Food Science and Engineering, Qilu University of Technology, Shandong Academy of Sciences, Jinan 250353, China

**Keywords:** extraction and purification, tunable aqueous polymer-phase impregnated resins, chromatography, capsaicin

## Abstract

Capsaicin, which mainly comes from pepper, exhibits anticancer, antioxidant, and anti-obesity properties. This work aims to construct a comprehensive technology for the extraction and purification of capsaicin from capsicum oleoresin. The tunable aqueous polymer phase impregnated HZ816 resins were selected in extraction step. In the extraction process, 3 g of impregnated HZ816 macroporous resin was employed per system. The results showed that a higher molecular weight of Polyethylene glycol (PEG) and 1-ethyl-3-methyl imidazolium acetate ([Emim] [OAc]) are more beneficial to the improvement of the yield of capsaicin. Screening experiment using fractional factorial designs indicated that the amount of sample loading, pH, and concentration of [Emim] [OAc] and PEG 6000 significantly affect the yield of capsaicin. Mathematical models of capsaicin yield in tunable aqueous polymer-phase impregnated resins were established and optimum condition was obtained using response surface methodology. The optimum impregnated phase was the polymer phase of an aqueous two-phase system which contained 18.5% (*w*/*w*) PEG6000, 15% (*w*/*w*) sodium citrate, and 10% (*w*/*w*) [Emim] [OAc] at pH 6.5. Under the optimal conditions, the yield of capsaicin reached 95.82% when the extraction system contains 0.25 g capsicum oleoresin. Ultimately, capsaicinoids extract was purified by reverse-phase resin (SKP-10-4300) chromatographic column. The capsaicin recovery and purity achieved 85% and 92%, respectively.

## 1. Introduction

The capsaicinoids family (for example capsaicin, nordihydrocapsaicin, dihydrocapsaicin, homodihydrocapsaicin, homocapsaicin) leads to intense spicy flavor of hot peppers, chilies, and some pungent foods [1]. The main components of the majority of capsicum species are capsaicin and dihydrocapsaicin [2,3]. The Food and Drug Administration (FDA) and European Medicines Agency (EMA) have approved capsaicin as a topical treatment for neuropathic pain, and pointed out that its analgesic effect depends on the dose [4]. At present, capsaicin also has some cases of application of topical analgesics for painful clinical diseases [5,6,7]. Oral capsaicin or direct consumption of pepper also has important pharmacological effects on human health. Moreover, natural capsaicin is also exploited owning to its anti-inflammation [8], anticancer [9,10,11], antibacterial, and antioxidant [12,13] properties. Furthermore, it is available for cardiovascular and gastrointestinal diseases [14]. The preparation technology of high-purity capsaicin has attract a strong interest of pharmaceutical chemists due to its wide biological activities.

A variety of techniques, such as aqueous enzymatic extraction [15], solvent extraction [16], and microwave- or ultrasound-assisted extraction [17,18] can be used for the extraction of capsaicin. However, these processes have obvious drawbacks, including large amounts of organic solvents, low recovery and purity, lengthy processes, etc. Furthermore, a purification step must complement to obtain a high-purity capsaicin product. Currently, the capsaicinoids extract can be purified by simulated moving bed chromatography [19], high-performance liquid chromatographic techniques [20], sequential centrifugal partition chromatography (SCPC) [21], and high-speed counter current chromatography (HSCCC) [22]. However, these purification technologies often represent the bottleneck of the whole production process because of high cost and sophisticated of those equipment.

For the above-mentioned reasons, aqueous two phase extraction technology combined with chromatography was established by our research group for preparation of high-purity capsaicin [23,24]. An aqueous two-phase system (ATPS) has been widely employed for purifying biological materials in biotechnology and biochemistry in the past decades. The high concentration of water (between 65% and 90% in mass) in these systems provides a mild and biocompatible environment for the extraction target molecules [25]. However, the low interfacial tension, small density differences, and high viscosities of the ATPS will result in long time consumption on phase separation [26,27]. Thus, phase separation might become a decisive procedure. In order to accelerate phase separation, the auxiliary equipment and extra energy are necessary [28]. Recently, the new ‘tunable aqueous polymer-phase impregnated resins’ (TAPPIR^®^)-technology, which was prepared by immobilizing one phase of ATPS in porous particles solved these challenges. In 2012, Schembecker et al. were approved the relevant patent (NO.-US 20140228549 A1) for this technology [29]. Recently, the researchers have conducted a preliminary discussion on the TAPPIR technology and obtained some scientific achievements [30,31,32,33,34,35]. The results demonstrated that the multistage processing of TAPPIR was excellent as a downstream processing technique. The polymer/salt ATPS is generally adopted and the polymer-rich phase is impregnated into porous solids due to its viscosity characteristics [31,32,33]. It is generally accepted that the PEG/citrate ATPSs are more beneficial to the extraction of bioactive substances [36]. Thus, the PEG/sodium citrate ATPSs were commonly used in the TAPPIR extraction step. According to the literature [37], the capsaicin can be extracted more efficiently from fresh chili by the pure 1-ethyl-3-methyl imidazolium hydrogen sulfate ([Emim] [HSO_4_]) and 1-ethyl-3-methylimidazolium acetate ([Emim] [OAc]). However, the high price and viscosity of the two ionic liquids (ILs) hinders the application of this technology at present. Therefore, a low quantity of two ILs was introduced in this work for their superior extraction ability to enhance the yield of the capsaicin. In addition to the extraction efficiency, the reusability of impregnated solids in multiple cycles also determines the competitiveness of TAPPIR [34,35].

In addition to the disadvantage of ATPS, the recovery of capsaicin was also reduced due to two steps chromatographic purification in our previous studies [23,24]. The further optimization of the extraction and purification method of capsaicin is very necessary. Practice shows that an inexpensive and novel reverse-phase resin (SKP-10-4300, Jinan, Shandong, China ) has an obvious advantage in the capsaicin purification process [24]. Hence, the combination TAPPIR and one-step chromatography technology was investigated and SKP-10-4300 was applied in this work.

The purpose of this work was to construct an efficient method for the separation and purification of capsaicin from capsicum oleoresin by combination of TAPPIR and chromatography. The effect of the relevant parameters such as the type impregnated resins and molecular weight of polymer, system pH, the amount of sample loading and addition of ionic liquid the capsaicin were investigated in the TAPPIR extraction step. Subsequently, the purification process of capsaicin was implemented using reverse phase chromatography. Adsorption and desorption properties of SKP-10-4300 was explored and a high recovery and purity of capsaicin were obtained.

## 2. Results and Discussion

### 2.1. Extraction of Capsaicin by TAPPIR^®^-Technology

#### 2.1.1. Selection of Macroporous Resins

During extraction experiment, the macroporous resins might absorb the capsaicin. The desorption capacities of the macroporous resins in the back-extraction step will directly affect the yield of capsaicin. In order to select the optimum macroporous resins, the static adsorption and desorption of different macroporous resins for capsaicin was investigated. The results are displayed in Table 1. It is obvious that the adsorption capacities of D101-I and HZ816 were higher than others. Their ratio of the static desorption were up to 100%. However, the price of HZ816 (26000 CNY per ton) was much cheaper than D101-I (45000 CNY per ton). The HZ816 macroporous resin is a better choice for the further experiment. In addition, the capsaicin yield of the TAPPIR system (impregnated HZ816 macroporous resin) was higher than that of static adsorption by HZ816 macroporous resin according to the preliminary experiment (data not shown). Therefore, HZ816 was selected as the impregnated resin for the following experiment.

#### 2.1.2. Effect of Ionic Liquid and PEG Molecular Weight (MW) on the Yield of Capsaicin in TAPPIR Technology

**Figure 1:**
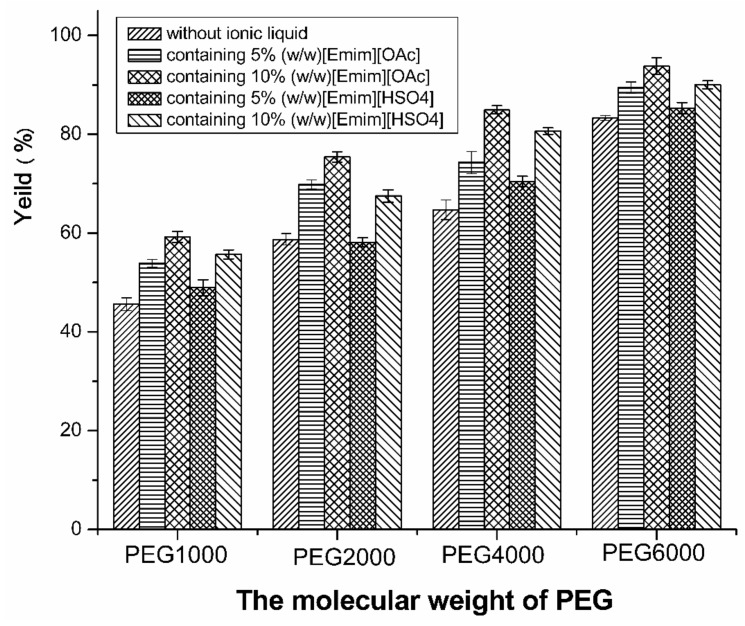
Ionic liquid and PEG molecular weight (MW) on extraction of capsaicin in TAPPIR technology. The HZ816 impregnated resin were prepared in 18% (*w*/*w*) PEG (1000, 2000, 4000 and 6000)/15% (*w*/*w*) sodium citrate top phase. The ionic liquid (10%, *w*/*w*) was added into the ATPSs when needed. The preliminary TAPPIR experiment was implemented at pH 7.0 and 25 °C. For each TAPPIR extraction system, 3 g impregnated resins were dispersed in 10 mL bottom phase of the ATPS with 0.2 g capsicum oleoresin.

To select the suitable ionic liquid extractant and PEG molecular weight, the capsaicin presented in capsicum oleoresin was extracted by TAPPIR technology at pH 7.0 and 25 °C. As shown in Figure 1, the capsaicin yield of the system without ionic liquid is generally lower than that of the system containing ionic liquid ([Emim] [OAc] or [Emim] [HSO_4_]). The attribution to imidazolium-based ILs is apt to enter into the PEG-rich phase which was the impregnation solution in TAPPIR technology, and that will be of benefit to improving the extraction efficiency of the hydrophobic biomolecules [38,39]. Meanwhile, [Emim] [OAc] was more favorable to the improvement of the capsaicin yield than [Emim] [HSO_4_]. It is attributed to the intrinsic basicity of pure [Emim] [OAc], which may contribute to the capsaicin extraction [37]. However, the extraction performance of [Emim] [OAc] was not investigated as its electrochemical behavior. On the other hand, the results show that the higher molecular weight of PEG is beneficial to the extraction of capsaicin. When the PEG molecular weight changed from 1000 to 6000, the yield of capsaicin was increased greatly. In this case, it can be proposed that the higher the hydrophobicity of the phase was, the higher yield of capsaicin was. Consequently, [Emim] [OAc] and PEG 6000 were used in the further experiments.

#### 2.1.3. Analysis of Screening Experiments

**Figure 2:**
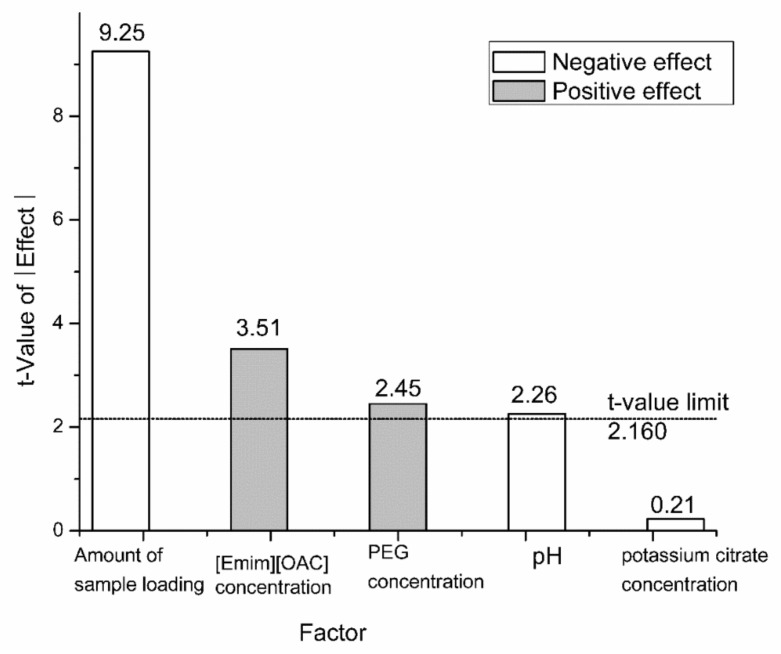
The influence of the main effects on the yield of capsaicin were analyzed with a Pareto chart. The line of the t-value limit is a reference. The factor has a significant contribution to the response as the *t*-value above the reference line (*p* < 0.05). Gray indicates that the effect of this factor is positive, and white indicates that the effect of this factor is negative.

As shown in Table 2, the fractional factorial design experiments were employed to screen the significant factors for extraction of capsaicin. It needs to specially point out that a small range of the pH (5.5–7.5) was set due to the stability of TAPPIR extraction system [33]. The Pareto chart, which shows the impact of the main factors on the response, can be created based on the results. As shown in Figure 2, the PEG concentration, [Emim] [OAC] concentration, pH, and sample loading have a statistically significant influence on the capsaicin yield (*p* < 0.05). The potassium citrate concentration had no remarkable influence on the extraction of capsaicin and was fixed at 15% (*w*/*w*) in the further experiment. The sequence of the significant main effects is the amount of sample loading > [Emim] [OAC] concentration > PEG concentration > pH. It is also displayed that the amount of sample loading is the most important variable in realizing optimum extraction condition. It will be a benefit to improving the capsaicin yield by decreasing the quantity of sample loading due to its negative effect. However, such experimental phenomena may be interpreted that the capsaicin had got saturation in the impregnated HZ816 macroporous resin. Effect of the amount of sample loading was also employed by single factor experiment using 3 g impregnated HZ816 macroporous resin during the extraction (data not shown). Based on comprehensive consideration of the single-factor experiment the 0.25 g capsicum oleoresin was added in subsequent studies.

#### 2.1.4. Steepest Ascent Experiment

In order to achieve the vicinity of optimization area, the steepest ascent experiment was carried out. A detailed design and the results are listed in Table 3. In this study, a maximum yield of 96.12% was obtained with system 6. The central point, which composed of 18.5% (*w*/*w*) PEG6000, 10% (*w*/*w*) [Emim] [OAC], and pH 6.5, was chosen for further experiments.

#### 2.1.5. Optimization of TAPPIR Technology Using RSM

To obtain the optimum extraction condition, a Box–Behnken design (BBD, Table 4) was employed in this work. Five center points were set to estimate the uncertainty variance which caused by the experimental process. The analysis of variance (ANOVA) results were shown in Table 5. The model F-value of 28.84 represents that the model is significant. It is also indicated that only a 0.01% probability of the model F-value being this large is able to appear due to noise. The “Prob> F” < 0.05 confirmed that model terms are significant. In this case, A (PEG6000 concentration), B ([Emim] [OAc] concentration), C (pH), BC, A^2^, B^2^, C^2^ are significant model terms. Compared with the pure error, the value of 3.56 means that the lack of fit is not significant. The coefficient of variation lower value of CV (1.54%) indicated that results of the experiment was greatly reliable. In summary, the quadratic models has an excellent applicability and can also be used directly. According to statistical calculations, the relation between the capsaicin yield and A, B, C was expressed as follows (in uncoded levels):

Y = − 136.72 + 19.57A − 0.207B + 18.61C + 0.001AB + 0.18AC + 0.38BC − 0.58A^2^ − 0.073B^2^ − 2.13C^2^ (1)

**Figure 3:**
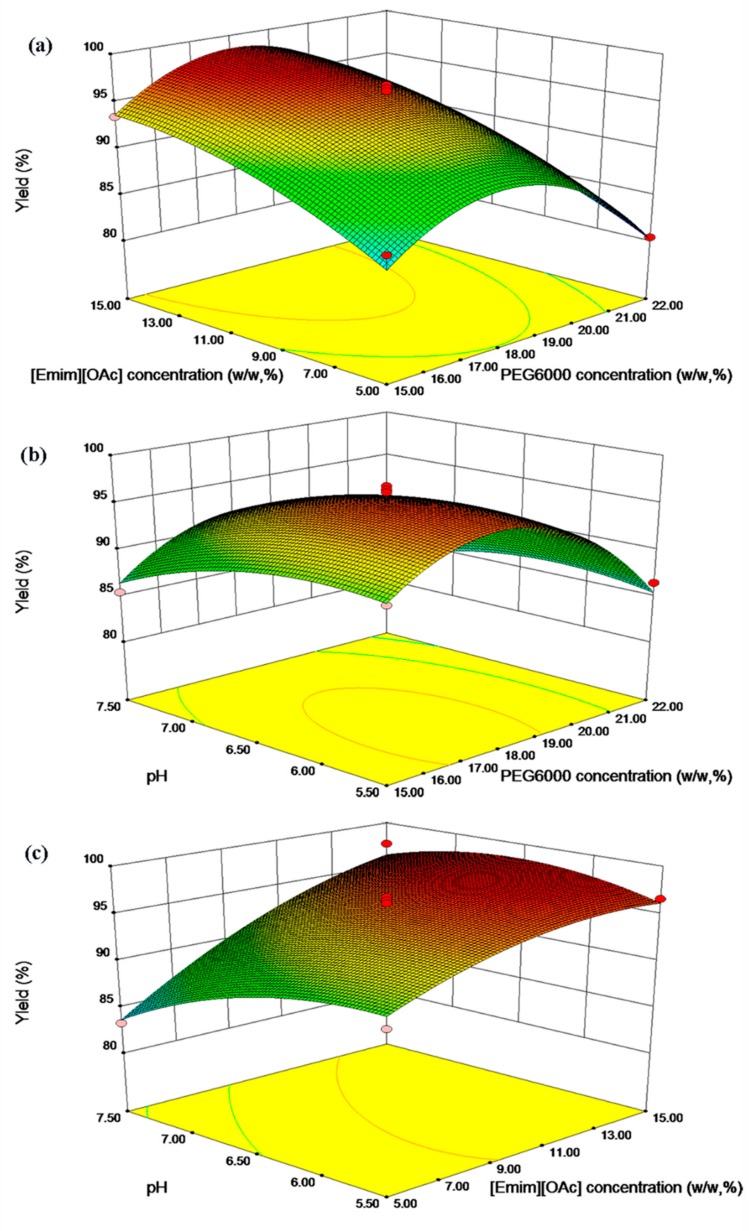
The response surface plot were shown as follows: (**a**) The effect of the concentration [Emim] [OAC] and PEG6000 on the response at pH 6.5. (**b**) The effect of the concentration of PEG6000 and pH on the response when the [Emim] [OAC] concentration was 10% (*w*/*w*). (**c**) The effect of the pH and concentration of [Emim] [OAC] on the response when the PEG6000 concentration was 18.5% (*w*/*w*).

The high values of R-squared (R^2^ = 0.974) and adjusted R-squared (R^2^_adj_ = 0.940) implies that a very good correlation exists between predicted and experimental data. The response surfaces obtained by displaying Equation (1) in three-dimensional plots are presented in Figure 3. It is clear that the response variable (Y) achieved the maximum at the ‘0’ level (A: 18.5% (*w*/*w*) PEG6000). The increasing of the capsaicin yield is obvious as the increasing of PEG6000 concentration in the beginning. It might be attributed to the fact that the phase in the HZ816 macroporous resins becomes more hydrophobic with increasing concentration of PEG6000. In other words, the more hydrophobic property of the phase in the HZ816 macroporous resins enhances the yield of the hydrophobic capsaicin. However, the steric hindrance effect might play a leading role and lead the capsaicin yield decreased when the PEG6000 concentration exceeded 18.5% (*w*/*w*).

Meanwhile, the concentration of [Emim] [OAC] has a positive effect on extraction performance of capsaicin. As shown in Figure 3a,c, the yield of capsaicin is increased with increasing the concentration of [Emim] [OAC] regardless of the high or low levels of other variables. As it is discussed in Section 3.2.2, this IL preferentially partitions to the impregnation systems, attracting capsaicin to this phase and leading to an increase in yield of capsaicin. The hydrophobicity of the impregnation systems was strengthened due to the presence of IL and the hydrophobic biomolecules were transferred to the impregnation system by the hydrophobic driving force [38].

Under alkaline conditions, the ionization of capsaicin tends to transfer from the more hydrophobic PEG-rich phase to the more hydrophilic salt-rich phase owing to its ionization action. Therefore, as shown in Figure 3b,c, the yield of capsaicin decreases in the impregnation system as the pH increases. A similar phenomenon was also discovered in ATPS extraction work [24]. This could be explained by the structure of the capsaicin molecule, which contains a phenolic hydroxyl group. In the alkaline condition, the H^+^ is easily ionized and generates the phenonium ion which is strongly hydrophilic and can easily be solved by water [40]. On the contrary, the capsaicin molecule was distributed to impregnated HZ816 macroporous resin system for its hydrophobicity in the acidic condition. This means that electrostatic interaction is not the main driving force in the extraction.

According to the RSM results, the highest extraction efficiency (95.82%) of the capsaicin was obtained when 0.25 g capsicum oleoresin was added in the system. Coincidentally, the optimum condition is just the central point of the RSM design. It is 3 g HZ816 macroporous resin impregnation systems which contains the top phase of 18.5% (*w*/*w*) of PEG6000/15% (*w*/*w*) sodium citrate with the addition of 10% (*w*/*w*) [Emim] [OAc] aqueous two phase system at pH 6.5. To confirm the predicted result, the comparative analysis should be done. As shown in Table 4, it is obvious that the average of experimental value (95.82%) of the five central points (runs of 3, 11, 13, 16, 17) is in full accord with the predicted value. It also indicates that the model equation can accurately predict the maximum capsaicin yield in the TAPPIR system.

#### 2.1.6. Extraction Performance of the Impregnation Resin

**Figure 4:**
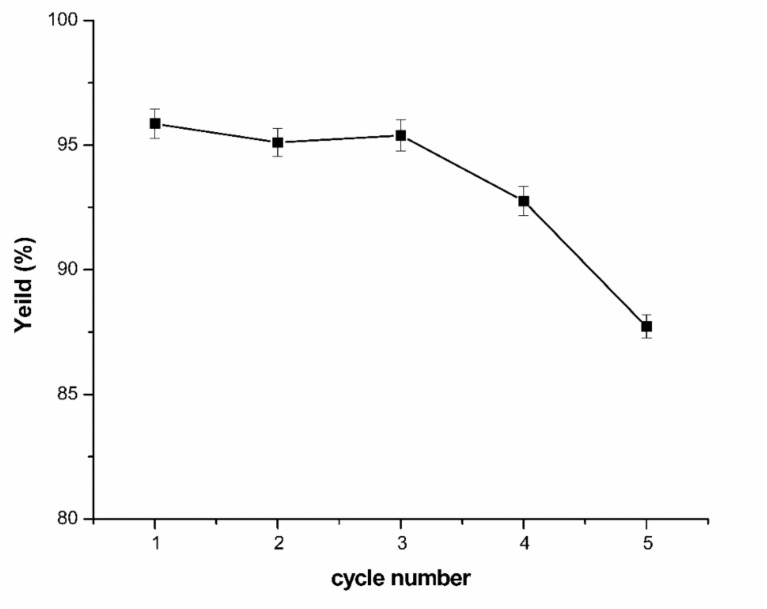
Extraction performance of the impregnation resin.

The stability of the impregnation of the PEG phase were demonstrated experimentally [33,35]. To estimate the extractive properties of the TAPPIR technology, five cycles were investigated based on the optimum condition. The results were illustrated in Figure 4. The yield of the first three cycles remained relatively constant. However, the yield of the capsaicin was reduced significantly after the fourth extraction process. In the circumstances, the equilibration of impregnation system should be reconstructed to enhance the extraction yield [35]. Therefore, the HZ816 impregnation resin should be equilibrated again after reusing four times to maintain a high capsaicin yield in this TAPPIR system.

### 2.2. Purification of Capsaicin by Reversed Chromatography

Compared with our previous work [23,24], high-purity capsaicin was obtained by a one-reverse-phase chromatographic step after the TAPPIR extraction in this work. The inexpensive SKP-10-4300 reverse resin was employed in this step. The loading solution were prepared according to Section 3.2.2 and Section 3.2.5 in this paper. The SKP-10-4300 reverse resin had a relatively larger static adsorption capacity (20 g/L) [24]. According to the previous studies [41,42], the purity of the product was determined by the ratio of height to diameter of the bed and the polarity of mobile phase. Therefore, based on the previous studies, the optimal operational conditions were determined as follows:

#### 2.2.1. Adsorption Capacities on SKP-10-4300 Resins

The different concentration and pH of sample loadings were prepared since the solubility of capsaicin increased with the pH increasing. The volume of the loading solution was kept constant at 150 mL. The designs and results were given in Table 6. Obviously, the dynamic adsorption capacity of the SKP-10-4300 to capsaicin is increased with the decrease in the pH of the sample loading. This is because the stronger acidity of the sample loading leads to the fact that the more capsaicin exists in molecular forms. Consequently, the interactions between the weak-polar capsaicin molecule and nonpolar SKP-10-4300 is strengthen, and vice versa. In other words, the lower pH of sample loading is, the higher adsorption capacity of the SKP-10-4300 to capsaicin is. On the other hand, the highest adsorption rate of 99.2% was obtained at the lowest concentration of the capsaicin pH of the loading solution. However, the decreased degree of adsorption rate was slight as the concentration and pH of the sample loading rises. In order to reduce the time consumption, the concentration of capsaicin and the pH sample loading were selected at 1.80 mg/mL and 8, respectively, in further experiments.

**Figure 5:**
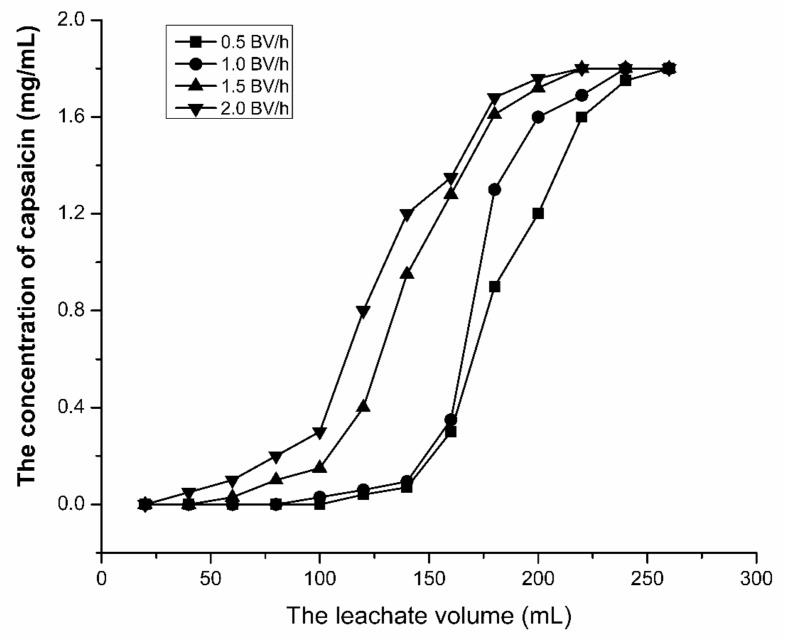
Effect of the sample loading flow-rate on the adsorption capacity of the SKP-10-4300 resin.

Based on the experiment above, the influence of the sample loading flow-rate was discussed. The leak curves were drawn and shown in Figure 5. From the leak curves (Figure 5), we can see that the sample loading flow-rate has great influence on the adsorption performance of capsaicin no to SKP-10-4300. While the sample loading flow-rate is up to 1.5 BV/h, the adsorption capacity is decreased significantly. It is attributed to the fact that the adsorption of weakly polar capsaicin on a non-polar porous structure of reversed-phase resin takes a certain amount of time. If the flow rate is too high, the target component is less likely to be adsorbed on the resin and is eluted by the loading solvent. However, it will take a long time to load the same volume of the loading solution at a low flow rate. Thus, considering both adsorption rate and production efficiency, the flow rate of the sample loading was finally selected to be 1.0 BV/h.

#### 2.2.2. Capsaicin Elution

**Figure 6:**
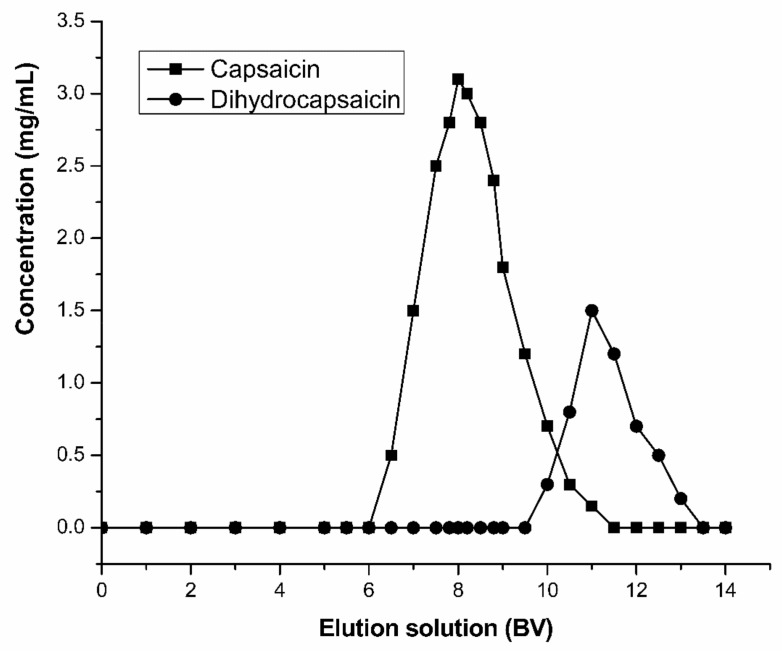
Effect of the elution solution on capsaicin separation. 0–2 BV: Distilled water; 2–4 BV:20% (*v*/*v*) ethanol/80% (*v*/*v*) distilled water; 4–6 BV: 45% (*v*/*v*) ethanol/55% (*v*/*v*) distilled water; 6–14 BV: 45% (*v*/*v*) ethanol/55% (*v*/*v*) aqueous sodium hydroxide (1%, *w*/*w*).

**Figure 7:**
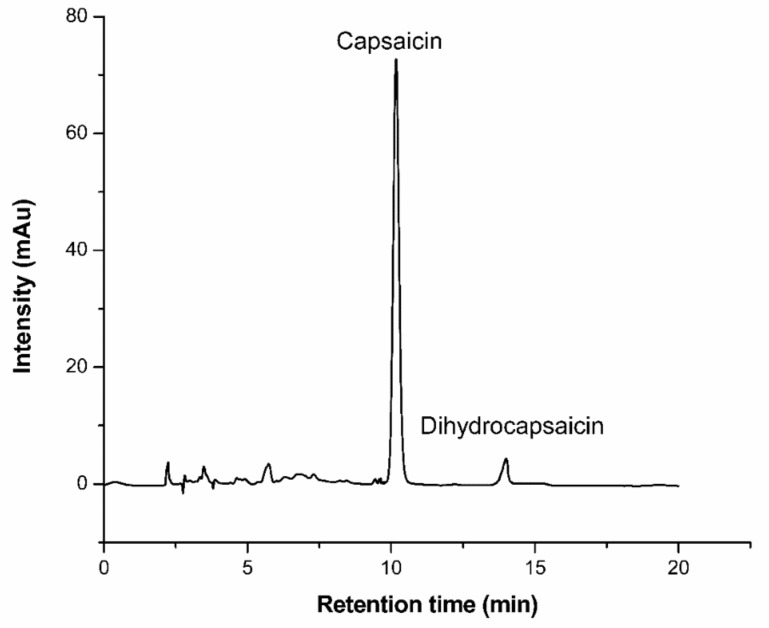
HPLC chromatograms of capsaicin eluent from the SKP-10-4300 resin.

After the reversed-phase resin adsorbed and balanced the capsaicin, the 2 BV deionized water was employed to remove the impurities which were not adsorbed on the reversed-phase resin. In order to improve the recovery and purity of the target product, a proper step-gradient elution was adopted in this work. Tthe NaOH solution (1%, *w*/*w*) was added in the elution solution to increase the sample desorption efficiency [1,42]. First, the effect of flow rate were investigated using elution solvent of 45% (*v*/*v*) ethanol/55% (*v*/*v*) aqueous sodium hydroxide (1%, *w*/*w*). Results of the experiments were illustrated in Table 7. When the flow rate of the elution is gradually increased, the desorption rate of capsaicin is gradually reduced. In addition, the low flow rate, which can increase the resolution of capsaicinoids, will also increase the recovery and purity of capsaicin. Nevertheless, the low flow rate causes an increase in the time taken, which directly leads to a decrease in the production efficiency of capsaicin. By comprehensive consideration, the flow rate of the elution was set at 1.0 BV/h in the next experiment. The optimized gradient elution conditions and results were shown in Table 8. The capsaicin recovery and purity were up to 85% and 92%, respectively. The dynamic desorption curve and the chromatograms of capsaicin eluent by HPLC are shown in Figure 6 and Figure 7, respectively. The main components were capsaicin and dihydrocapsaicin in the eluate. It is confirmed that the SKP-10-4300 resin has been successfully applied to the separation of capsaicin.

## 3. Materials and Methods

### 3.1. Materials

Capsicum oleoresin was provided by Yingchaolabeier Co., Ltd. (Shandong, China). The capsaicin standard was purchased from Sigma (St. Louis, MO, USA). Macroporous adsorption resins (MARs) D101-1, 201 × 7, HZ202, D301, D301G, D201, and ADS-17 were provided by Hebei Huazhong Chemical Industries Co., Ltd. (Hebei, China). HZ816, HZ835, and HZ915MARs were purchased from Shanghai Huazhen Technology Co., Ltd. (Shanghai, China). The reverse-phase resin (SKP-10-4300) was obtained from Jinan Bona Biological (Jinan, Shandong, China). [Emim] [HSO_4_] and [Emim] [OAc] were prepared by Lanzhou Greenchem ILS, LICP. CAS. China (Lanzhou, Gansu, China). Polyethylene glycol (PEG1000, PEG2000, PEG4000, PEG6000) was obtained from Shanghai Lingfeng. Sodium citrate were acquired from Tianjin Guangcheng Chemical Company (Tianjin, China). Methanol (MeOH) of HPLC grade, ethyl acetate, and ethanol were purchased from Sinopharm Chemical Reagent Co., Ltd. (Shanghai, China). The others reagents were analytical grade. Deionized water was employed throughout in the experiment.

### 3.2. Methods

#### 3.2.1. Static Adsorption and Desorption Tests of the MARs

All the MARs require pretreatment to remove impurities trapped inside the pores. The resins were soaked in ethanol for 48 h and washed by deionized water until the wash was clear. Then the resins were soaked in 4–5% (*w*/*w*) HCl solution for 4 h, and washed by deionized water till the washes was neutral. Then the resins were immersed in 4–5% (*w*/*w*) NaOH solution for 4 h, and washed by deionized water till the washing liquid was neutral. Then, the MARs were preserved in deionized water and removed from the deionized water by vacuum drawing and filtering system for static adsorption and desorption test. A total of 0.2 g of various resins was accurately weighed and mixed with 50 mL capsaicin standard liquid (44.2 μg/mL) in a conical flask. The experiment was carried out at 30 °C in constant temperature oscillator with 100 r/min rotational speed for 24 h. Then, the resins which have adsorbed the capsaicin were rinsed twice by deionized water. Furthermore, 50 mL of ethyl acetate was added to desorb the capsaicin. The original alternative resins were estimated by the ratio of adsorption and desorption.

#### 3.2.2. The Procedure of the TAPPIR^®^ Extraction

In this work, the experimental procedure for TAPPIR extraction was implemented as described by Winssen et al. [31]. Firstly, the ATPSs were prepared and centrifuged at 1500 r/min at temperature for 10 min. The pH value was adjusted by adding B-R buffer which was prepared by adding amount of 0.20 M NaOH to the solution including 0.04 H_3_PO_4_, 0.04 M CH_3_COOH, and 0.04 M H_3_BO_3_. Then, the selected resin was impregnated into the top phase of the ATPS to construct impregnation resin. Impregnation took place in an ultrasonic bath for 1h. After impregnation, the particles were separated from the dispersion by filtration and sipped up the surface with sterile filter paper. Subsequently, 3 g of these impregnated particles were dispersed in 10 mL bottom phase of the ATPS with capsicum oleoresin. This dispersion was shaken at 25 °C in constant temperature oscillator with 180 r/min rotational speed for 1 h. After this, the impregnated resin containing capsaicinoids was removed and the back extraction was carried out using ethyl acetate to accurately determine the amount of capsaicin. During the back extraction, the system was shaken at 25 °C in a constant temperature oscillator with a 180 r/min rotational speed for 30 min. The capsaicin content of the ethyl acetate phase was determined using HPLC. The capsaicin yield is defined by Equation (2) as follows:

(2) $$Y=\;\frac{C_{\mathrm{ethyl}\;\mathrm{acetate}}\times V_{\mathrm{ethyl}\;\mathrm{acetate}}}{M_{\mathrm{added}}\times C_{\mathrm{added}}}$$

where *C*_ethyl acetate_, *V*_ethyl acetate_, *M*_added_, and *C*_added_ are represent the capsaicin concentration in ethyl acetate phase, the ethyl acetate phase volume, the added quality of capsaicin oleoresin, and the capsaicin concentration in capsaicin oleoresin, respectively.

#### 3.2.3. Response Surface Methodology (RSM)

Design Expert version 8.0.6.1 (Stat-Ease Inc., Minneapolis, MN, USA) software was employed in the process of experiment design, analysis, and modeling.

Screening experiment was initially performed through fractional factorial designs. The concentration of PEG6000, concentration of potassium citrate, concentration of [Emim] [OAC], pH, and amount of sample loading were taken into account in this step. The capsaicin yield (Y) was set as the response variable.

In order to obtain an optimized extraction system and evaluate the interaction between various factors, the response surface methodology (RMS) was introduced. The optimum conditions can be predicted by the second-order model as follows:

(3) $$Y=\beta_{0}+\overset{k}{\underset{i=1}{\sum }}\beta_{i}X_{i}+\overset{k}{\underset{i=1}{\sum }}\beta_{ii}X_{i}^{2}+\sum \underset{i<j}{\sum }\beta_{ij}X_{i}X_{j}+\varepsilon$$

Among them, Y is the response variable (in percentage), *β*_0_ is the fixed response value of the experimental center point, *β_i_*, *β_ij_*, and *β_ii_* were the linear, cross product coefficients, and quadratic, respectively, and *X_i_* and *X_j_* were independent variables of the code.

#### 3.2.4. Preparation of the Reversed Chromatographic Column

The reversed resins should be soaked in ethanol for 12 h to make the resin full swelling. The preparative reversed phase column (10 mm × 800 mm) was filled with SKP-10-4300 reverse-phase resin (43 mL) with a length of 55 cm. Then, the glass chromatography column was sequentially leached with ethanol, 50% (*v*/*v*) ethanol, 25% (*v*/*v*) ethanol, and 10% (*v*/*v*) ethanol over 1 h. The flow rate of the eluent was 2 bed volume (BV) per hour.

#### 3.2.5. Preparation of the Reversed Chromatographic Loading Solution

After TAPPIR extraction, the impregnated resin containing capsaicinoids was removed. The back-extraction step was employed as in Section 3.2.2 in this paper. Then, the ethyl acetate phase was separated and concentrated under reduced pressure at 40–50 °C using a rotary evaporator. Subsequently, the concentrate was diluted to appropriate concentration of capsaicin solution by adding 10% ethanol solution with different pH. The pH of ethanol solution was adjusted by 4–5% (*w*/*w*) HCl or NaOH solution.

#### 3.2.6. HPLC Analyses

The capsaicin concentration of samples were determined by HPLC on an Agilent 1260 infinity LC system at 35 °C. The EC-C18 column (Agilent Poroshell 120, 4.6 × 150 mm, 5 μm) (made in USA) was employed in the analysis test. The detection wavelength of the DAD detector was set at 280 nm. The mobile phase was a mixture (V_methanol_:V_water_ = 70:30) with a flow rate of 0.5 mL/min.

## 4. Conclusions

The results have confirmed that the combination of TAPPIR technology and reverse chromatography as a downstream processing technique is a feasible approach for extracting and purifying capsaicin from the capsicum oleoresin. The HZ816 resin was selected in the TAPPIR extraction step based on the static adsorption and desorption experiment. The tunable aqueous polymer phases were the top phase of PEG/sodium citrate aqueous two-phase system which contained [Emim] [OAc] as adjuvants. The sequence of the significant main effects is the amount of sample loading > [Emim] [OAC] concentration > PEG concentration > pH. The optimum impregnated phase was the polymer phase of aqueous two phase system which contained 18.5% (*w*/*w*) PEG6000, 15% (*w*/*w*) sodium citrate, and 10% (*w*/*w*) [Emim] [OAc] at pH 6.5. Under the optimal conditions, the yield of capsaicin reached up to 95.82% when the extraction system contained 0.25 g capsicum oleoresin using 3 g impregnated HZ816 macroporous resin. Furthermore, the capsaicinoid extract was purified by a reverse-phase resin (SKP-10-4300) chromatographic column. The capsaicin recovery and purity achieved 85% and 92%, respectively. The new combined technology has enormous potentiality and may be applied to large-scale industrial production of capsaicin. Further investigations will focus on the crystallization of capsaicin in our lab.

## Figures and Tables

**Table 1:** The ratios of adsorption and desorption capsaicinoids on macroporous resins in static state.

Type	Static Absorption Rate“ (%)	Static Desorption Rate (%)
D101-I.	94.65	100
HZ816	95.2	100
HZ915	35.09	88.76
HZ835	82.94	100
ADS -17	84.19	100
D201	52.14	57.31
201 × 7	56.07	74.57
D301	39.85	69.74
D301G	38.14	51.49
HZ202	79.62	98.34

**Table 2:** (Y) Capsaicin in TAPPIR technology using fractional factorial design.

Runs	Concentration of PEG6000 (*w*/*w*, %)	Concentration of Sodium Citrate (*w*/*w*, %)	Concentration of [Emim] [OAc] (*w*/*w*, %)	pH	Sample Loading (g)	Y (yield, %)
1	15	18	15	5.5	0.3	74.36
2	21	18	5	7.5	0.1	88.72
3	15	18	5	7.5	0.3	58.94
4	21	12	15	5.5	0.3	79.22
5	15	12	15	5.5	0.1	96.17
6	21	18	15	5.5	0.1	98.56
7	21	18	5	5.5	0.3	72.34
8	15	12	5	5.5	0.3	65.13
9	21	18	15	7.5	0.3	75.45
10	15	12	5	7.5	0.1	81.38
11	18	15	10	6.5	0.2	91.01
12	18	15	10	6.5	0.2	92.64
13	15	18	5	5.5	0.1	87.75
14	18	15	10	6.5	0.2	90.07
15	21	12	5	5.5	0.1	95.36
16	15	18	15	7.5	0.1	92.78
17	15	12	15	7.5	0.3	67.72
18	21	12	15	7.5	0.1	95.24
19	21	12	5	7.5	0.3	65.67

**Table 3:** The detailed design and results of the ascent experiment.

Runs	Concentration of PEG6000 (*w*/*w*, %)	Concentration of [Emim] [OAc] (*w*/*w*, %)	pH	Y (yield, %)
1	15	5	7.5	79.23
2	15.7	6	7.3	82.54
3	16.4	7	7.1	85.86
4	17.1	8	6.9	89.12
5	17.8	9	6.7	93.33
6	18.5	10	6.5	96.12
7	19.2	11	6.3	96.02
8	19.9	12	6.1	95.87
9	20.6	13	5.9	94.13
10	21.3	14	5.7	93.24
11	22	15	5.5	90.58

**Table 4:** The yield of capsaicin in TAPPIR technology using a Box–Behnken design.

Runs	A: Concentration of PEG6000 (*w*/*w*, %)	B: Concentratio of [Emim] [OAc] (*w*/*w*, %)	C: pH	Y (yield, %)
1	18.5	15	5.5	96.60
2	15	10	7.5	85.45
3	18.5	10	6.5	96.15
4	15	5	6.5	86.35
5	15	15	6.5	93.45
6	22	15	6.5	87.51
7	22	10	5.5	86.50
8	22	5	6.5	80.34
9	22	10	7.5	83.21
10	18.5	5	7.5	83.27
11	18.5	10	6.5	96.43
12	18.5	15	7.5	97.59
13	18.5	10	6.5	96.79
14	15	10	5.5	91.25
15	18.5	5	5.5	90.04
16	18.5	10	6.5	95.36
17	18.5	10	6.5	94.38

**Table 5:** Statistical evaluation for the results of the Box–Behnken design.

Source	Sum of Squares	Degrees of Freedom	Mean Square	F value	Prob > F
Model	504.70	9	56.08	28.84	0.0001
A-A	44.84	1	44.84	23.06	0.0020
B-B	154.44	1	154.44	79.41	<0.0001
C-C	27.64	1	27.64	14.21	0.0070
A-B	1.225 × 10^−3^	1	1.225 × 10^−3^	6.299 × 10^−4^	0.9807
A-C	1.58	1	1.58	0.81	0.3981
B-C	15.05	1	15.05	7.74	0.0272
A^2^	211.71	1	211.71	108.86	<0.0001
B^2^	13.92	1	13.92	7.16	0.0317
C^2^	19.08	1	19.08	9.81	0.0166
Total	518.31	16			
Lack of fit	9.91	3	3.30	3.56	0.1257
CV%	1.54				
R^2^	0.974				
Adjusted R^2^	0.940				

**Table 6:** Effect of the concentration and pH of sample loadings.

pH	Concentration of Capsaicin in Sample Loading (mg/mL)	Absorption Capacity (mg/mL)	Absorption Rate (%)
6	0.91	11.1	99.2
7	1.23	10.8	98.4
8	1.8	10.2	96.2
9	2.08	8.9	91.8

The sample was loaded into the glass column by a peristaltic pump at a flow rate of 0.5 BV/h at room temperature.

**Table 7:** Effect of the eluent flow rate using elution solvent of 45% (*v*/*v*) ethanol/55% (*v*/*v*) aqueous sodium hydroxide (1%, *w*/*w*).

Eluent Flow Rate (BV/h)	Desorption Rate (%)	Recovery of Capsaicin (%)	Purity of Capsaicin (%)	Elution Time (h)
0.5	99	51	66	6.8
1	96	46	63	3.5
1.5	94	38	55	2.3
2	93	30	42	1.7

**Table 8:** The optimized gradient elution conditions and results.

Step	Eluent	Recovery of Capsaicin (%)	Purity of Capsaicin (%)
1	Distilled water 2 BV		
2	20% (*v*/*v*) ethanol/80% (*v*/*v*) Distilled water 2 BV		
3	45% (*v*/*v*) ethanol/55% (*v*/*v*) Distilled water 2 BV	85	92
4	45% (*v*/*v*) ethanol/55% (*v*/*v*) aqueous sodium hydroxide (1%, *w*/*w*) 8 BV		

The type and height of column was 10 mm × 800 mm and 55 cm, respectively.
